# Growth differentiation factor 15 is not modified after weight loss induced by liraglutide in South Asians and Europids with type 2 diabetes mellitus

**DOI:** 10.1113/EP091815

**Published:** 2024-07-04

**Authors:** Carlijn A. Hoekx, Maaike E. Straat, Maurice B. Bizino, Huub J. van Eyk, Hildebrandus J. Lamb, Johannes W. A. Smit, Ingrid M. Jazet, Saskia C. A. de Jager, Mariëtte R. Boon, Borja Martinez‐Tellez

**Affiliations:** ^1^ Division of Endocrinology, Department of Medicine Leiden University Medical Center Leiden The Netherlands; ^2^ Einthoven Laboratory for Experimental Vascular Medicine Leiden University Medical Center Leiden The Netherlands; ^3^ Department of Radiology Leiden University Medical Center Leiden The Netherlands; ^4^ Department of Medicine Radboud University Medical Center Nijmegen The Netherlands; ^5^ Laboratory of Translational Immunology University Medical Centre Utrecht Utrecht The Netherlands; ^6^ Department of Nursing Physiotherapy and Medicine, SPORT Research Group (CTS‐1024), CERNEP Research Center University of Almería Almería Spain; ^7^ Biomedical Research Unit Torrecárdenas University Hospital Almería Spain; ^8^ CIBER de Fisiopatología de la Obesidad y Nutrición (CIBEROBN) Instituto de Salud Carlos III Granada Spain

**Keywords:** ethnic differences, metabolic diseases, obesity, weight‐reducing drugs

## Abstract

**Abstract:**

Glucagon‐like peptide‐1 receptor (GLP‐1R) agonists induce weight loss in patients with type 2 diabetes mellitus (T2DM), but the underlying mechanism is unclear. Recently, the mechanism by which metformin induces weight loss could be explained by an increase in growth differentiation factor 15 (GDF15), which suppresses appetite. Therefore, we aimed to investigate whether the GLP‐1R agonist liraglutide modifies plasma GDF15 levels in patients with T2DM. GDF15 levels were measured in plasma samples obtained from Dutch Europids and Dutch South Asians with T2DM before and after 26 weeks of treatment with daily liraglutide (*n* = 44) or placebo (*n *= 50) added to standard care. At baseline, circulating GDF15 levels did not differ between South Asians and Europids with T2DM. Treatment with liraglutide, compared to placebo, decreased body weight, but did not modify plasma GDF15 levels in all patients, or when data were split by ethnicity. Also, the change in plasma GDF15 levels after treatment with liraglutide did not correlate with changes in body weight or HbA_1c_ levels. In addition, the dose of metformin used did not correlate with baseline plasma GDF15 levels. Compared to placebo, liraglutide treatment for 26 weeks does not modify plasma GDF15 levels in Dutch Europid or South Asian patients with T2DM. Thus, the weight loss induced by liraglutide is likely explained by other mechanisms beyond the GDF15 pathway.

**Highlights:**

- **What is the central question of this study?**
  Growth differentiation factor 15 (GDF15) suppresses appetite and is increased by metformin: does the GLP‐1R agonist liraglutide modify plasma GDF15 levels in patients with type 2 diabetes mellitus (T2DM)?
- **What is the main finding and its importance?**
  Plasma GDF15 levels did not differ between South Asians and Europids with T2DM and were not modified by 26 weeks of liraglutide in either ethnicity. Moreover, there was no correlation between the changes in plasma GDF15 levels and dosage of metformin administered, changes in body weight or HbA1c levels. The appetite‐suppressing effect of liraglutide is likely exerted via pathways other than GDF15.

## 1 INTRODUCTION

The number of people living with type 2 diabetes mellitus (T2DM) has rapidly increased globally in recent years and is expected to continue to rise (Reed et al., 2021). Obesity is a major risk factor for the development of T2DM (Kopelman, 2000). South Asian ethnicity is another well‐known risk factor, as South Asians have a significantly higher risk of developing T2DM at a younger age and a lower body mass index (BMI) than other ethnic groups, including Europids (Flowers et al., 2019). The underlying mechanism for this increased risk of developing T2DM in South Asians is not entirely known; however, it is likely multifactorial. Their disadvantageous body composition, consisting of a higher fat mass percentage with more visceral adipose tissue and a lower muscle mass than Europids, is a significant contributing factor to a higher insulin resistance state (Bakker et al., 2013; Eastwood et al., 2015; McKeigue et al., 1991). Hormonal cues may also play a role in the increased risk of developing T2DM in South Asians. Differences in appetite‐regulating hormones such as glucagon‐like peptide‐1, leptin and ghrelin have previously been described in South Asians compared to other ethnicities (Benedetti et al., 2019; Mente et al., 2010; Singh, 2015). These hormonal variations could further contribute to the increased risk of South Asians developing T2DM.

Metformin is the first‐line pharmacological treatment for patients with new‐onset T2DM. It acts by reducing hepatic gluconeogenesis, enhancing peripheral insulin sensitivity, and increasing the secretion of the gut hormone GLP‐1, ultimately leading to reduced plasma glucose levels. Besides improving glucose regulation, metformin also reduces body weight (Diabetes Prevention Program Research Group, 2012; Lee & Morley, 1998). Recent translational studies from two independent research groups have demonstrated that metformin increases circulating growth differentiation factor 15 (GDF15) levels to suppress appetite, thereby inducing weight loss (Coll et al., 2020; Day et al., 2019). GDF15 is a stress‐induced cytokine and a member of the transforming growth factor‐β superfamily (Lee et al., 2017). It induces satiety by binding to the glial cell line‐derived neurotrophic factor (GDNF) family receptor α‐like (GFRAL) located in the area postrema and solitary tract of the hindbrain. GFRAL subsequently interacts with the tyrosine kinase co‐receptor RET, which induces phosphorylation of the signalling molecules AKT, extracellular signal‐regulated kinases 1 and 2 (ERK1/2), and phospholipase C, thereby inducing anorexia (Dong & Xu, 2024; Emmerson et al., 2017; Hsu et al., 2017; Ling et al., 2023; Mullican et al., 2017; Yang et al., 2017). In addition, GDF15 induces satiety by signalling through satiety‐inducing cholecystokinin neurons located in the hindbrain (Dong & Xu, 2024; Worth et al., 2020). Clinical trials involving individuals with overweight and obesity who were treated with a long‐acting GDF15 receptor analogue showed a reduction in food intake and body weight, supporting the weight‐reducing effect of GDF15 (Benichou et al., 2023). Beyond appetite suppression, GDF15 also contributes to weight loss by enhancing energy expenditure, at least in mice (Dong & Xu, 2024; Wang et al., 2023). Accordingly, metformin also increases energy expenditure in preclinical studies, which was mechanistically at least in part through enhancing activation of energy‐combusting brown adipose tissue (Geerling et al., 2014; Ziqubu et al., 2023).

Despite its effectiveness, metformin monotherapy is insufficient to maintain glucose regulation in some patients with T2DM (Kahn et al., 2006). Therefore, additional therapeutic strategies have been developed to improve glycaemic parameters, reducing the risk of T2DM‐associated complications. One of these therapies is GLP‐1 receptor (GLP‐1R) agonism, which mimics the effects of the incretin hormone GLP‐1 to stimulate glucose‐dependent insulin secretion and reduce glucagon secretion, both contributing to its glucose‐lowering effects (Bizino et al., 2021; Perreault et al., 2021). In addition, GLP‐1R agonism has been shown to induce weight loss in patients with T2DM to a greater extent than metformin (Lazzaroni et al., 2021; Liu et al., 2017), and its weight loss effect is partly attributed to its ability to promote satiety (Drucker, 2018).

Unlike metformin, the underlying mechanism of appetite suppression due to GLP‐1R agonism is not entirely known. GLP‐1 receptors are expressed throughout the hindbrain, including the area postrema and nucleus of the solitary tract where GLP‐1 receptor agonism induces appetite suppression. Of note, these regions also harbour GFRAL receptors, which mediate the effects of GDF15 in inducing satiety and reducing food intake, as mentioned above (Cork et al., 2015; Frikke‐Schmidt et al., 2019). This spatial overlap in receptor expression raises the question of whether GLP‐1R agonism may modulate the GFRAL/GDF15 signalling pathways and thereby contribute to its appetite‐suppressing and weight‐reducing effects.

Understanding the mechanism involved in appetite suppression following GLP‐1R agonism is of great significance, considering its increasing popularity as a treatment for both diabetes and obesity. Furthermore, with approximately 20% of the world's population of South Asian descent and considering their markedly increased risk of developing obesity and type 2 diabetes compared to subjects of Europid descent, studying potential differences in the GFRAL/GDF15 system in South Asians compared to Europids as a potential underlying mechanism is relevant as well.

Therefore, in the current study, we aimed to study (1) whether circulating GDF15 levels differ between Dutch South Asian and Dutch Europid patients with T2DM; (2) whether the GLP‐1R agonist liraglutide modifies plasma levels of GDF15 in either ethnicity; and (3) whether changes in GDF15 levels are related to the reduction in body weight after liraglutide treatment in either ethnicity.

## 2 METHODS

### 2.1 Participants and study design

#### 2.1.1 Participants

This study is a secondary analysis of two previously performed double‐blind, placebo‐controlled, randomized clinical trials that were both designed to study the effect of treatment with liraglutide for 26 weeks on glucose regulation and cardiovascular endpoints in patients with overweight and obesity and T2DM (Bizino et al., 2019; van Eyk et al., 2019) and performed at the Leiden University Medical Center (LUMC). In total, 50 patients of Dutch Europid (hereinafter: ‘Europid’) origin (study 1) (Bizino et al., 2019) and 47 participants of Dutch South Asian (hereinafter: ‘South Asian’) origin (study 2) (van Eyk et al., 2019) were included. South Asian ethnicity is defined as having four grandparents who originally descended from Surinam, Bangladesh, India, Nepal, Pakistan, Afghanistan, Bhutan or Sri Lanka. Inclusion criteria were males and females aged 18–69 years, BMI ≥ 25 kg/m^2^, and HbA_1c_ levels of 7.0%–10.0% (53–86 mmol/mol) despite the use of metformin and/or sulfonylurea derivatives and/or insulin. General exclusion criteria were the use of other glucose‐lowering medication than those mentioned above, a history of renal or hepatic disease, surgery, pancreatitis, pregnancy or lactation, and the presence of any contra‐indication for magnetic resonance imaging (MRI). Both trials were performed between 2013 and 2018.

#### 2.1.2 Study approval

Both studies were performed by the principles of the *Declaration of Helsinki* (Association, 2013) and approved by the local ethics committee of the Leiden University Medical Center, Leiden, the Netherlands. All participants provided written informed consent before participation. The trials were registered at ClinicalTrials.gov (registration no. NCT01761318 and NCT02660047).

#### 2.1.3 Treatment regimen

After inclusion, all participants were randomized to receive daily treatment with liraglutide (Victoza®, Novo Nordisk A/S, Bagsværd, Denmark) or placebo (provided by Novo Nordisk A/S) with 1:1 stratification for sex and insulin use. At baseline, the dose of liraglutide was 0.6 mg per day (administered subcutaneously), which was titrated in two steps in 3 weeks to the maximum amount of 1.8 mg once daily. The dose was reduced, if necessary, in case of adverse events. During the study, participants were contacted weekly by telephone to assess adverse events and to discuss glucose management. During the entire 26 weeks, regular treatment options for optimal glycaemic control and regulation of cholesterol levels and blood pressure were given according to current clinical guidelines. Two Europid participants discontinued treatment. One of these participants was in the liraglutide group and discontinued treatment due to repeated hypoglycaemic events and was later diagnosed with type 1 diabetes mellitus. The other participant was in the placebo group and could not continue treatment because he was in detention. There were no serious adverse events related to study drug use, and adverse events reported were mild gastrointestinal problems (i.e., nausea and vomiting).

#### 2.1.4 Study design

Extensive descriptions of the design of both trials have been published elsewhere (Bizino et al., 2019; van Eyk et al., 2019). In short, at baseline and after 26 weeks of treatment, all included participants arrived at the outpatient clinic after at least a 6‐h fast. First, body weight, body composition and lean body mass were assessed by bioelectrical impedance analysis (BIA; scale Bodystat 1500, Bodystat Ltd, Douglas, UK). Then, venous blood samples were collected, and the participants underwent an MRI and proton magnetic resonance spectroscopy (^1^H‐MRS) to measure subcutaneous, visceral, epicardial and paracardial adipose tissue volume.

#### 2.1.5 Blood collection

After the collection of venous blood samples, serum and plasma were obtained by centrifugation and stored in the freezer at −80°C until analysis. Serum levels of total cholesterol, high‐density lipoprotein cholesterol (HDL‐C), and low‐density lipoprotein cholesterol (LDL‐C) were measured on a Modular P800 analyser (Roche Diagnostic, Mannheim, Germany). Due to logistical reasons, HbA_1c_ was initially measured with boronate‐affinity high‐performance liquid chromatography (Primus Ultra; Siemens Healthcare Diagnostics, Breda, the Netherlands) and later with ion‐exchange high‐performance liquid chromatography (Tosoh G8, Sysmex Nederland B.V., Etten‐Leur, the Netherlands). To ensure accurate and consistent results, HbA_1c_ levels obtained from the boronate affinity method were corrected based on the correlation coefficient obtained from validation samples measured on both analysers. Plasma GDF15 levels were measured with human magnetic bead‐based multiplex for the Luminex platform (LXSAHM; R&D systems, Minneapolis, MN, USA) according to the manufacturer's protocol.

#### 2.1.6 MRI

At baseline and after 26 weeks of treatment with liraglutide or placebo, participants underwent an MRI in the supine position, using a 3.0 tesla MRI scanner (Ingenia, Philips Healthcare, Best, the Netherlands) to assess epicardial and paracardial adipose tissue as well as visceral and abdominal adipose tissue volumes, as extensively described previously (van Eyk et al., 2019).

### 2.2 Statistical analyses

Data are expressed as means ± standard deviation. Data normality was confirmed using the Shapiro–Wilk test, visual histograms, and Q–Q plots. Baseline characteristics were compared between the treatment groups and ethnicities using the chi‐square test for binary values (i.e., sex and use of diabetes medication), an independent Student's *t*‐test, and the Mann–Whitney *U*‐test for normality distributed data. Non‐normally distributed data were log10 transformed (e.g., baseline creatine, baseline subcutaneous adipose tissue, visceral adipose tissue, subcutaneous/visceral adipose tissue ratio, paracardial adipose tissue, total cholesterol, and LDL‐C). Non‐parametric tests were performed on data that were non‐normally distributed after log10 transformation (e.g., diabetes duration, baseline fat percentage, baseline HbA_1c_ and metformin dose at baseline). To study the difference between circulating GDF15 levels between ethnicities, an independent *t*‐test was performed with log10 transformed data of baseline GDF15 levels to follow a normal distribution. A delta (Δ; 26‐week treatment minus baseline value) was created for every outcome. To study the effect of liraglutide on plasma GDF15 levels, body weight and HbA_1c_ an analysis of covariance (ANCOVA) was performed, adjusting for baseline values. Moreover, to examine the association of baseline and Δ plasma GDF15 levels with Δ body weight, Δ HbA_1c_, metformin dose, Δ glucose, Δ total cholesterol, Δ adipose tissue deposition and Δ kidney function, a non‐parametric Spearman's rank correlation (rho) was applied. All statistical analyses were performed using the SPSS Statistics v.25.0 (IBM Corp., Armonk, NY, USA), whereas all graphs were created with GraphPad Prism software version 9.3.1 for Windows (GraphPad Software, Boston, MA, USA). Significance was set at *P* < 0.05.

## 3 RESULTS

### 3.1 Baseline characteristics

At baseline, no significant differences in age, BMI, sex distribution, or other cardiometabolic parameters were observed between participants receiving liraglutide or placebo in either Europid or South Asian individuals with T2DM, as described previously for both trials (Bizino et al., 2019; van Eyk et al., 2019) (Supporting information Table S1). However, waist circumference (*P* = 0.029), paracardial (*P* = 0.013) and pericardial (*P* = 0.039) adipose tissue volumes were higher in participants from the liraglutide group than those from the placebo group at baseline when both ethnicities were combined (van Eyk et al., 2019). When comparing the baseline characteristics between Europid and South Asian individuals, age (*P* = 0.013), body weight (*P* < 0.001), body length (*P* < 0.001), BMI (*P* = 0.001), waist circumference (*P* < 0.001), waist to hip ratio (*P* = 0.001), visceral (*P* = 0.007), paracardial (*P* < 0.001), and pericardial adipose tissue volumes (*P* < 0.001), total cholesterol (*P* = 0.003), LDL‐C (*P* = 0.022) and metformin dose (*P* = 0.031) were significantly higher in Europids compared to South Asians. Conversely, South Asians had a longer duration of T2DM compared to the Europids (Table 1).

**TABLE 1:** Baseline characteristics.

	Europids		South Asians		Combined	
	Placebo (*n *= 25)	Liraglutide (*n *= 22)	Placebo (*n *= 25)	Liraglutide (*n *= 22)	Placebo (*n *= 50)	Liraglutide (*n *= 44)
Demographics						
Females (*n* (%))	11 (44)	9 (41)	14 (56)	14 (64)	25 (50)	23 (52)
Age (years)	58.9 ± 6.7	59.9 ± 6.4	54.6 ± 9.4	55.2 ± 11.1	56.7 ± 8.4	57.5 ± 9.2
Diabetes duration (years)	10.5 ± 6.9	10.4 ± 5.0	17.0 ± 9.8^#^	18.8 ± 10.3^##^	13.8 ± 9.0	14.6 ± 9.0
Clinical parameters						
Body weight (kg)	93.8 ± 12.9	98.6 ± 14.1	77.8 ± 12.4^###^	81.9 ± 11.0^###^	85.8 ± 14.9	90.3 ± 15.1
Body length (cm)	172.4 ± 9.7	173.4 ± 7.8	164.8 ± 9.4^##^	164.3 ± 8.1^###^	168.6 ± 10.2	168.9 ± 9.1
BMI (kg/m^2^)	31.5 ± 3.5	32.8 ± 4.3	28.6 ± 4.0^##^	30.4 ± 3.8	30.1 ± 4.0	31.6 ± 4.2
Waist circumference (cm)	108.4 ± 8.1	111.9 ± 9.6	98.4 ± 10.1^###^	104.1 ± 7.8*^,##^	103.4 ± 10.4	108.0 ± 9.5*
Hip circumference (cm)	106.2 ± 6.7	108.5 ± 8.5	104.0 ± 9.0	104.3 ± 7.1	105.1 ± 7.9	106.4 ± 8.0
Waist to hip ratio	1.0 ± 0.1	1.0 ± 0.1	0.9 ± 0.1^##^	1.0 ± 0.1*	1.0 ± 0.1	1.0 ± 0.1
Body fat percentage (%)	36.7 ± 9.0	36.7 ± 10.1	36.9 ± 9.8	37.2 ± 8.4	36.8 ± 9.3	37.0 ± 9.2
Subcutaneous adipose tissue (cm^2^)	330 ± 109	367 ± 143	326 ± 141	316 ± 97	328 ± 125	341 ± 123
Visceral adipose tissue (cm^2^)	200 ± 62	211 ± 88	149 ± 49^##^	187 ± 57*	174 ± 61	199 ± 74
Visceral/subcutaneous adipose tissue ratio	0.7 ± 0.3	0.7 ± 0.4	0.5 ± 0.3	0.7 ± 0.3	0.6 ± 0.3	0.7 ± 0.3
Epicardial adipose tissue (cm^2^)	9.6 ± 4.1	8.9 ± 4.4	9.1 ± 2.7	10.4 ± 3.2	9.3 ± 3.4	9.6 ± 3.9
Paracardial adipose tissue (cm^2^)	20.6 ± 10.0	25.8 ± 11.2	9.0 ± 4.5^###^	12.3 ± 4.4*^,###^	14.4 ± 9.5	19.1 ± 10.8*
Pericardial adipose tissue (cm^2^)	30.2 ± 12.3	34.7 ± 13.7	18.2 ± 5.6^###^	22.7 ± 6.5*^,##^	23.9 ± 11.1	28.7 ± 12.2*
HbA_1c_ (mmol/mol)	64.6 ± 10.3	66.5 ± 11.7	70.5 ± 12.1	64.8 ± 9.7	67.5 ± 11.5	65.7 ± 10.7
Total cholesterol (mmol/L)	4.8 ± 1.0	4.9 ± 1.0	4.5 ± 1.1	4.0 ± 0.7^##^	4.6 ± 1.1	4.4 ± 1.0
HDL‐C (mmol/L)	1.3 ± 0.4	1.2 ± 0.3	1.2 ± 0.3	1.2 ± 0.3	1.3 ± 0.3	1.2 ± 0.3
LDL‐C (mmol/L)	2.5 ± 0.9	2.6 ± 0.9	2.2 ± 1.0	2.0 ± 0.7^##^	2.4 ± 1.0	2.4 ± 0.9
Diabetes medication						
Metformin use (*n* (%))	25 (100)	22 (100)	23 (92)	22 (100)	48 (96)	44 (100)
Metformin (mg/day)	1982 ± 553	2093 ± 700	1728 ± 643	1750 ± 665	1860 ± 605	1922 ± 697
Sulfonylurea (*n* (%))	8 (32)	6 (27)	5 (20)	3 (14)	13 (26)	9 (21)
Insulin use (*n* (%))	16 (64)	14 (64)	19 (76)	17 (77)	35 (70)	31 (71)

*Note*: Adapted from the original data from treatment in Europids (Bizino et al., 2019) and South Asians (van Eyk et al., 2019). Two Europids were not included in the analyses since they discontinued treatment. Data are presented as means ± standard deviation. Asterisks (*) indicate significant differences between treatments within a specific ethnicity, and hash signs (^#^) indicate significant differences between ethnicities within a specific treatment group. *P < 0.05, ^#^ *P* < 0.05, ^##^ *P* < 0.01, ^###^ *P* < 0.001. Abbreviations: BMI, body mass index; HbA_1c_, haemoglobin A1c; HDL‐C, high‐density lipoprotein‐cholesterol; LDL‐C, low‐density lipoprotein‐cholesterol.

### 3.2 Liraglutide reduces body weight but not HbA_1c_ levels

As previously published, 26 weeks of liraglutide treatment, as compared to placebo, decreased body weight in both Europids (−4.3 ± 3.8 vs. +0.1 ± 2.5 kg*; P* < 0.001) (Bizino et al., 2019) and South Asians (−3.9 ± 3.6 vs. −0.6 ± 2.2 kg; *P* < 0.001) (van Eyk et al., 2019). The reduction in body weight induced by liraglutide was not different between ethnicities (*P* = 0.287). In addition, as previously published, 26 weeks of liraglutide treatment, as compared to placebo, did not decrease HbA_1c_ in either Europids (−11.6 ± 11.1 vs. −7.7 ± 9.4 mmol/mol; *P* = 0.265) (Bizino et al., 2019) or South Asians (−8.5 ± 11.2 vs. −6.8 ± 9.3 mmol/mol; *P* = 0.156) (van Eyk et al., 2019).

### 3.3 Liraglutide does not modify plasma GDF15 levels

**FIGURE 1:**
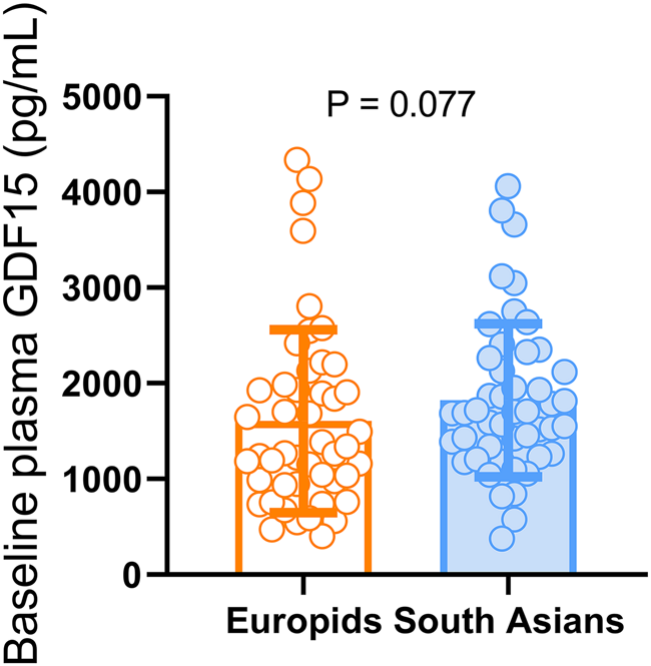
Comparison of plasma GDF15 levels in Europids and South Asians at baseline. Box plots showing plasma GDF15 levels in Europids (*n* = 47; orange box with circles) compared to South Asians (*n* = 47; blue box with circles) at baseline combined for the placebo and liraglutide treatment groups. Dots represent individual values, boxes represent means and error bars represent standard deviation (SD).

Next, we assessed plasma GDF15 levels at baseline and after 26 weeks of liraglutide treatment and ethnic differences herein. At baseline, plasma GDF15 levels were similar between the liraglutide and placebo groups in both Europids (1495 ± 838 vs. 1702 ± 1056 pg/mL; *P* = 0.651) and South Asians (1834 ± 790 vs. 1814 ± 825 pg/mL; *P* = 0.774). We combined the baseline GDF15 levels from the participants assigned to the liraglutide and placebo groups to increase the sample size. We observed that baseline levels of GDF15 were not statistically different in South Asians compared to Europids (1823 ± 800 vs. 1605 ± 956 pg/mL; *P* = 0.077, Figure 1). Additionally, we performed sensitivity analyses by splitting the data by sex but did not observe significant differences (data not shown).

**FIGURE 2:**
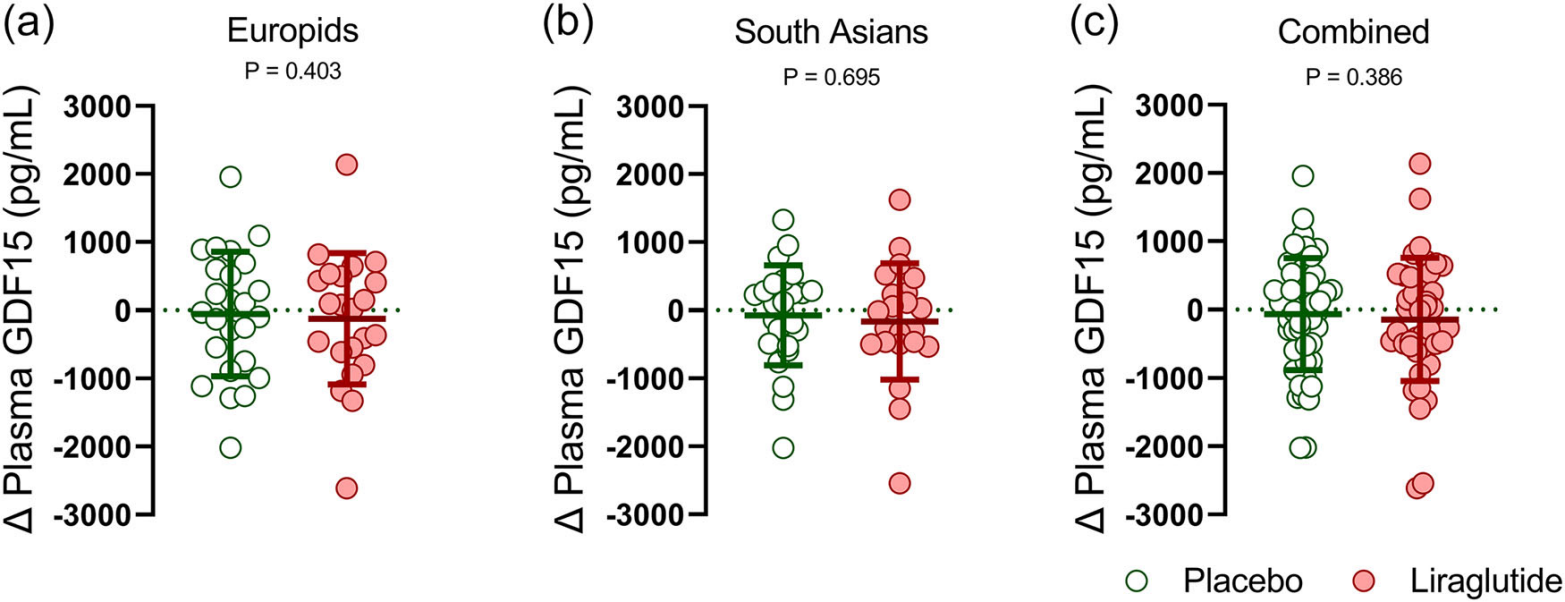
Changes in plasma GDF15 levels in Europids, South Asians and both ethnicities combined after 26 weeks of placebo or liraglutide treatment. Scatter plots showing changes in plasma GDF15 (26‐week treatment minus baseline values) of Europids (a), South Asians (b), and both ethnicities combined (c) after treatment with placebo (*n* = 50; green box and circles) or liraglutide (*n* = 44; red box and circles). Dots represent individual values, horizontal lines represent means and error bars represent standard deviation (SD).

**FIGURE 3:**
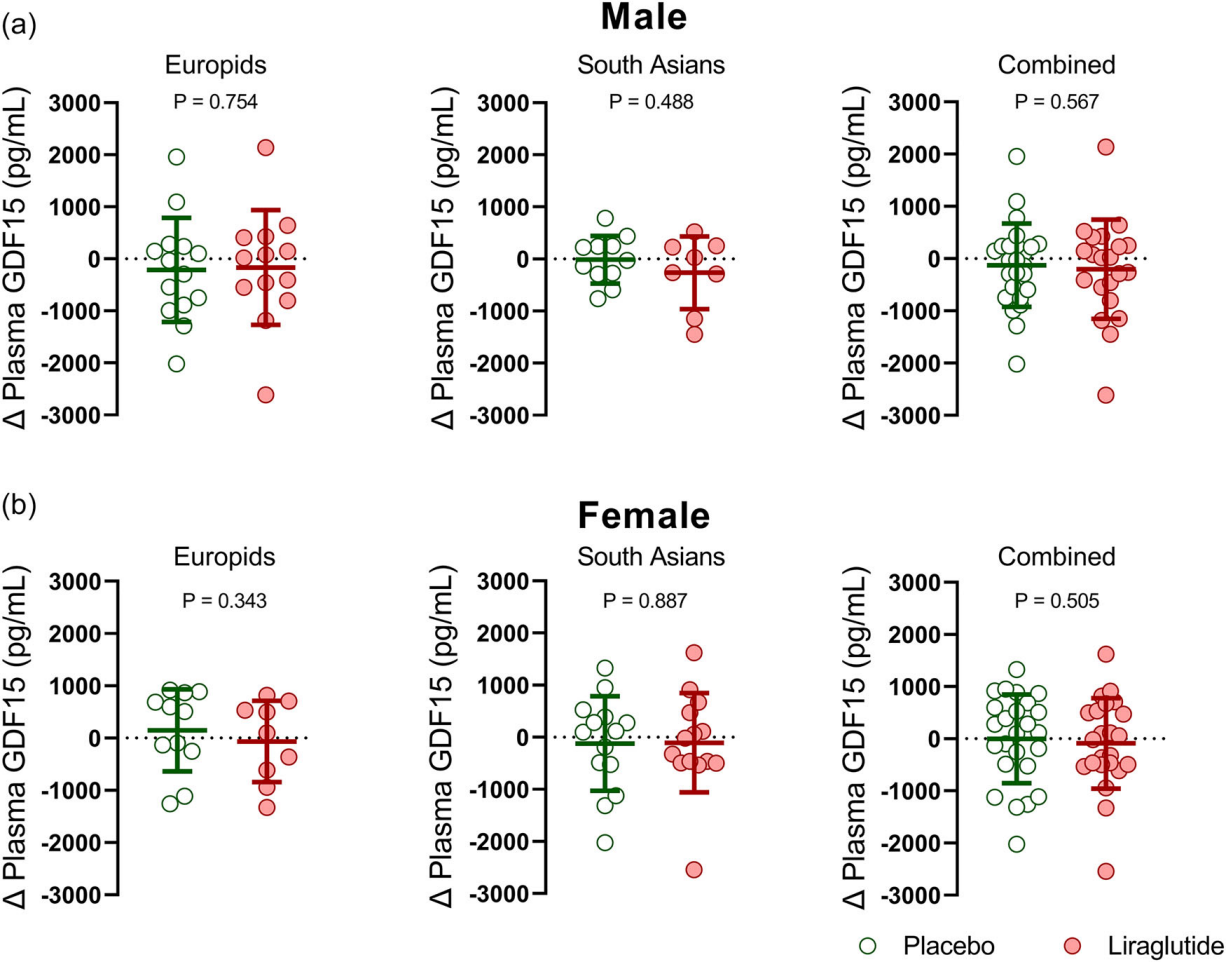
Changes in plasma GDF15 levels in male and female Europids, South Asians and both ethnicities combined after 26 weeks of placebo or liraglutide treatment. Scatter plots showing changes in plasma GDF15 levels (26‐week treatment minus baseline values) of (a) Europid males (placebo: *n* = 14; green circles; liraglutide: *n* = 13; red circles), South Asian males placebo: *n* = 11; green circles; liraglutide: *n* = 8; red circles) and males of both ethnicities combined after placebo (*n* = 25; green circles) or liraglutide (*n* = 21; red circles), and (b) Europid females (placebo: *n* = 11; green circles; liraglutide: *n* = 9; red circles), South Asian females (placebo: *n* = 14; green circles; liraglutide: *n* = 14; red circles) and females of both ethnicities combined after placebo (*n* = 25; green circles) or liraglutide (*n* = 23; red circles). Dots represent individual values, horizontal lines represent means and error bars represent standard deviation (SD).

Twenty‐six weeks of treatment with liraglutide, as compared to placebo, did not affect the change (i.e., 26 weeks minus baseline) in plasma GDF15 levels in either Europids (−124 ± 962 vs. −55 ± 911 pg/mL; *P* = 0.403, Figure 2a) or South Asians (−163 ± 853 vs. −74 ± 732 pg/mL; *P* = 0.695, Figure 2b). To increase the statistical power, we combined the data from both ethnicities. However, we still did not find significant differences in plasma GDF15 levels between the placebo and liraglutide groups (−144 ± 899 vs. −64 ± 818 pg/mL vs. liraglutide; *P* = 0.386, Figure 2c). Additionally, we repeated all analyses separately for men and women, and for men and women with different ethnicities and the lack of effect also persisted (Figure 3a,b).

### 3.4 Changes in plasma GDF15 levels do not relate to liraglutide‐induced decreases in body weight

**FIGURE 4:**
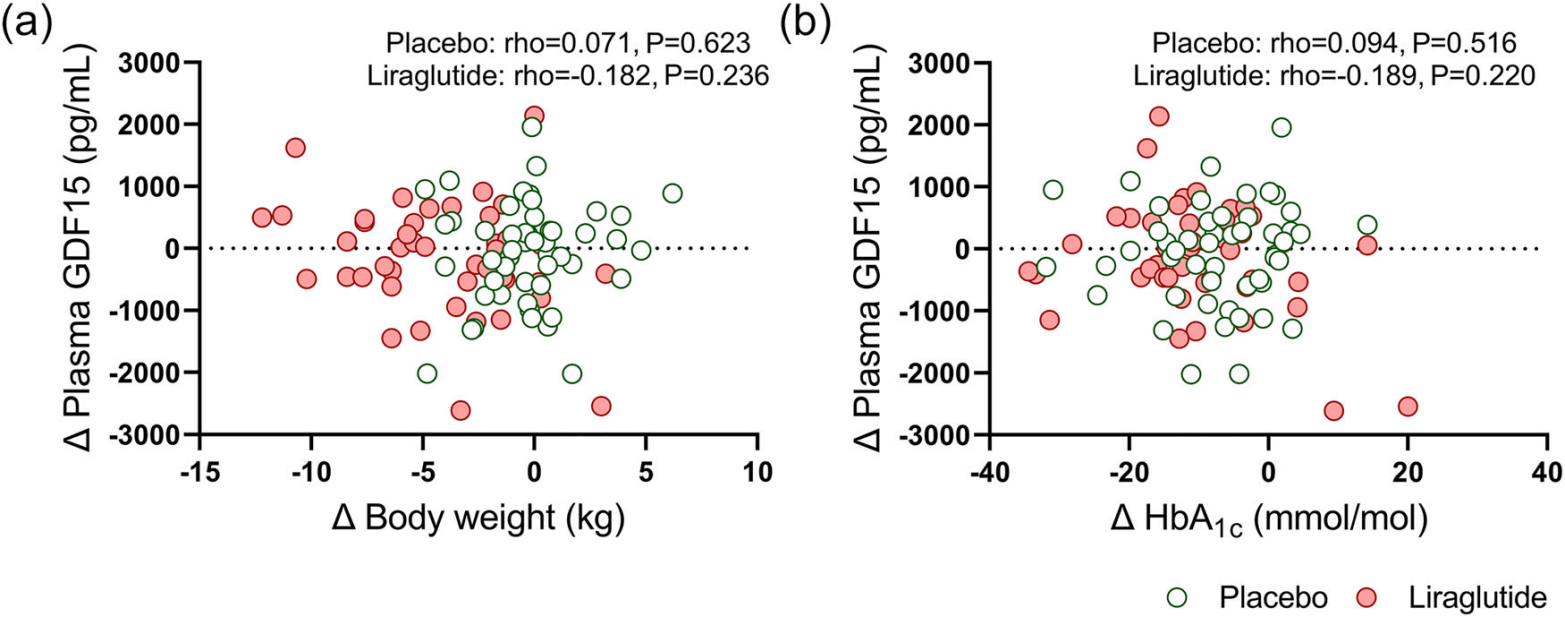
Correlations between changes in body weight and HbA_1c_ levels and changes in plasma GDF15 levels after 26 weeks of treatment with placebo or liraglutide. Spearman correlation, in both Europids and South Asians, combined, between the change of body weight and the change in plasma GDF15 levels (a) and change in plasma HbA_1c_ levels and change in plasma GDF15 levels (b) after treatment with placebo (*n* = 50; green circles) or liraglutide (*n* = 44; red circles). Dots represent individual values.

**FIGURE 5:**
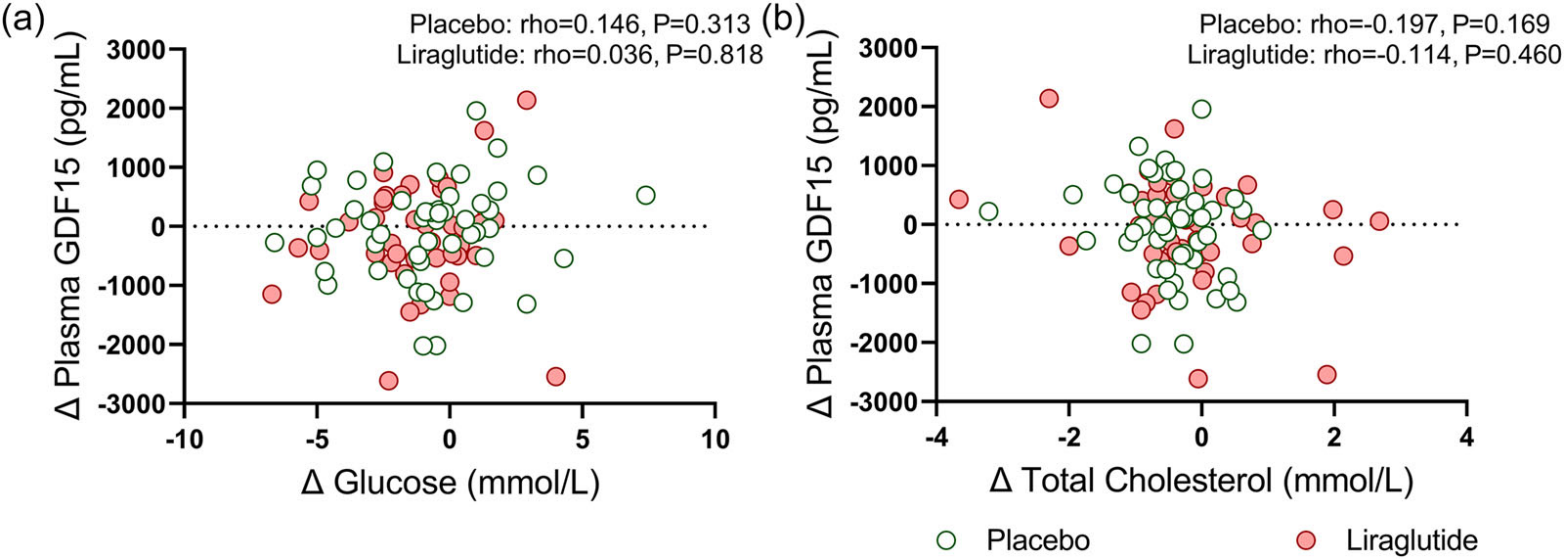
Correlations between changes in metabolic parameters and changes in plasma GDF15 levels after 26 weeks of treatment with placebo or liraglutide. Spearman's correlation plots between changes in fasting plasma glucose and changes in plasma GDF15 levels (a) and changes between total cholesterol levels and changes in plasma GDF15 levels (b) in both ethnicities combined after treatment with placebo (*n* = 50; green circles) or liraglutide (*n* = 44; red circles). For one participant in the placebo treatment group, the fasting glucose at the end of the study was missing (*n* = 43). Dots represent individual values.

**FIGURE 6:**
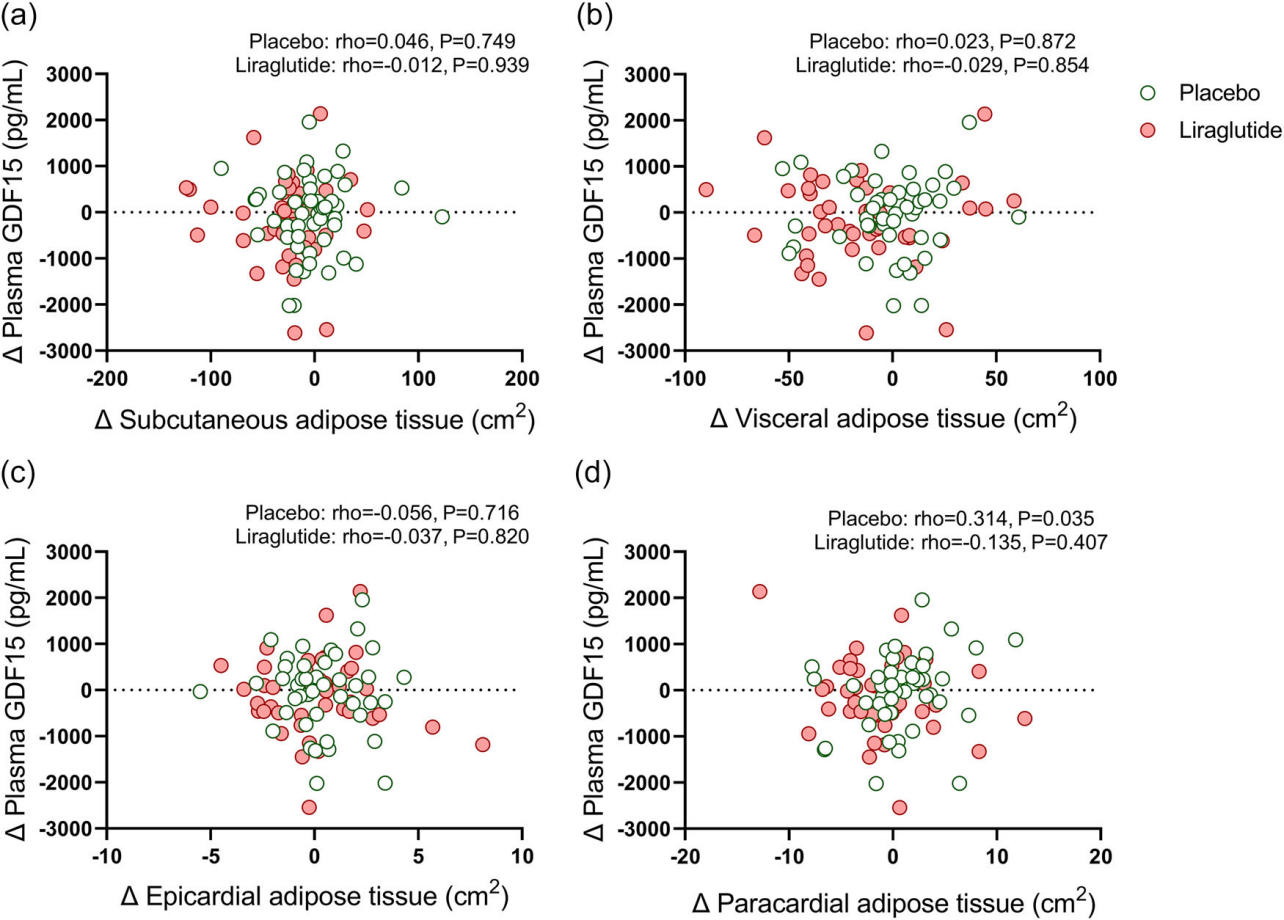
Correlations between changes in subcutaneous adipose tissue, visceral adipose tissue, epicardial adipose tissue and paracardial adipose tissue volumes and changes in plasma GDF15 levels after 26 weeks of treatment with placebo or liraglutide. Spearman's correlation plots between changes in subcutaneous adipose tissue (a), visceral adipose tissue (b), epicardial adipose tissue (c), or paracardial adipose tissue volumes (d) and the change of plasma GDF15 levels in both ethnicities combined after treatment with placebo (*n* = 50; green circles) or liraglutide (*n* = 44; red circles). For six participants in the placebo treatment group, the epicardial adipose tissue (*n* = 44) and for five participants in the placebo group the paracardial adipose tissue (*n* = 45) was not reported. For four participants in the liraglutide treatment group, both epicardial adipose tissue and paracardial adipose tissue volumes could not be reported (*n* = 40) at either the start of the study, or at the end of the study, or both. This was due to either unsuccessful ^1^H‐MRS of the heart due to low signal‐to‐noise ratio incorrect peak frequency, or due to missing data (Bizino et al., 2020; van Eyk et al., 2019). Dots represent individual values.

**FIGURE 7:**
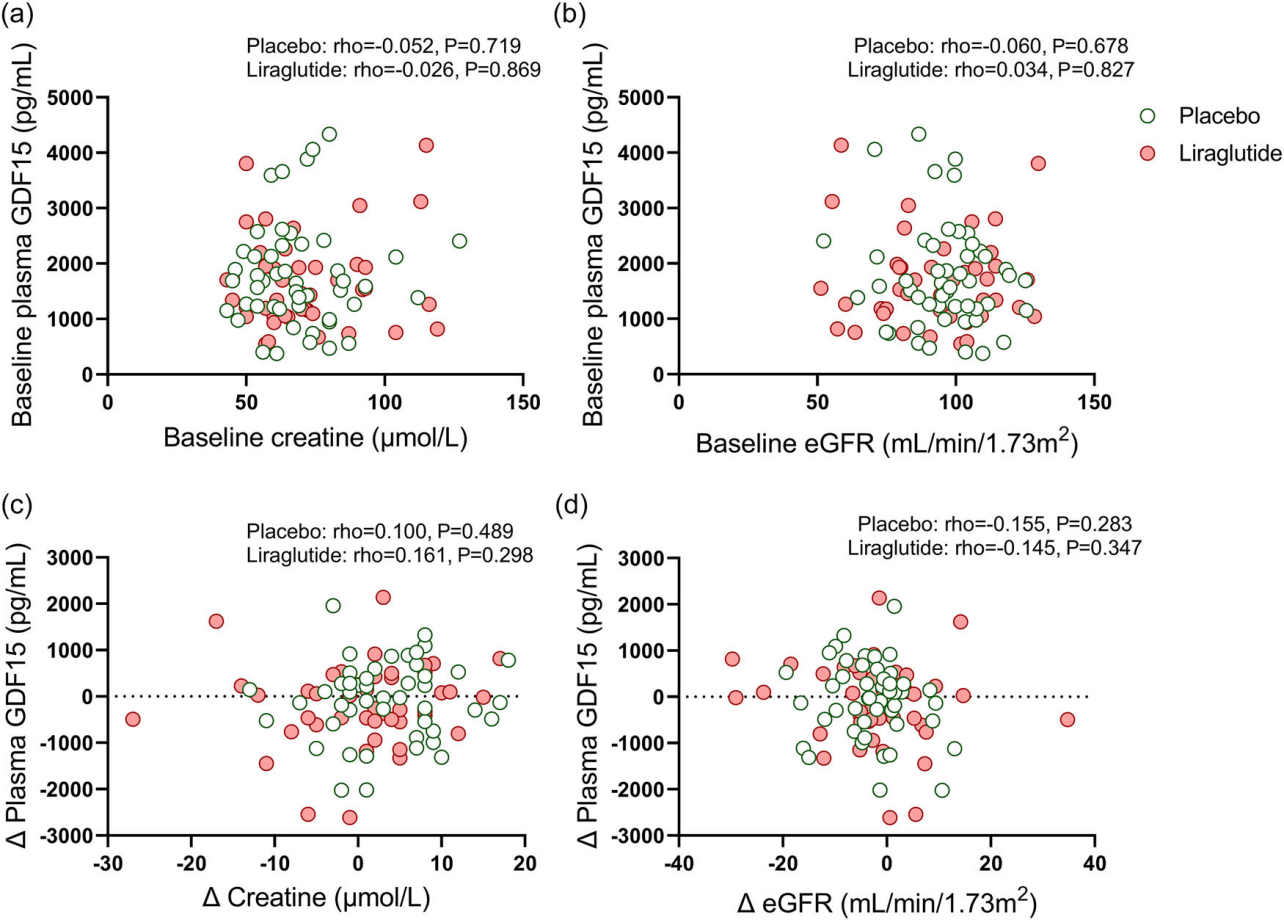
Correlations between markers for kidney function and plasma GDF15 levels at baseline and after 26 weeks of treatment with placebo or liraglutide. (a, b) Spearman's correlation plots between baseline serum creatinine levels (a) or estimated glomerular filtration rate (eGFR) calculated based on serum creatine levels (b) and baseline plasma GDF15 levels in both ethnicities combined. (c, d) Spearman's correlations between changes in plasma creatine levels (c) or changes in eGFR (d) and changes in plasma GDF15 levels in both ethnicities combined after placebo (*n* = 50; green circle) or liraglutide (*n* = 44; red circles). Dots represent individual values.

We next assessed whether the changes in body weight were related to changes in plasma GDF15 levels. Since we did not find any statistical interaction of ethnicity or sex on the changes in plasma GDF15 levels (*P* ≥ 0.5, data not shown), we pooled all data together to enhance statistical power for the subsequent analyses. After 26 weeks of treatment with liraglutide, as compared to placebo, changes in plasma GDF15 levels were not related to changes in body weight (rho = −0.182; *P* = 0.236 vs. rho = 0.071; *P* = 0.623, Figure 4a) or changes in HbA_1c_ levels (rho = −0.189; *P* = 0.220 vs. rho = 0.094; *P* = 0.516 Figure 4b). Additionally, we observed that changes in plasma GDF15 levels were not related to changes in blood parameters related to glucose and lipid metabolism (e.g., glucose or total cholesterol) and subcutaneous, visceral, epicardial, paracardial adipose tissue volumes or markers for kidney function (Figures 5, 6, 7).

### 3.5 Plasma GDF15 levels do not correlate with metformin doses

**FIGURE 8:**
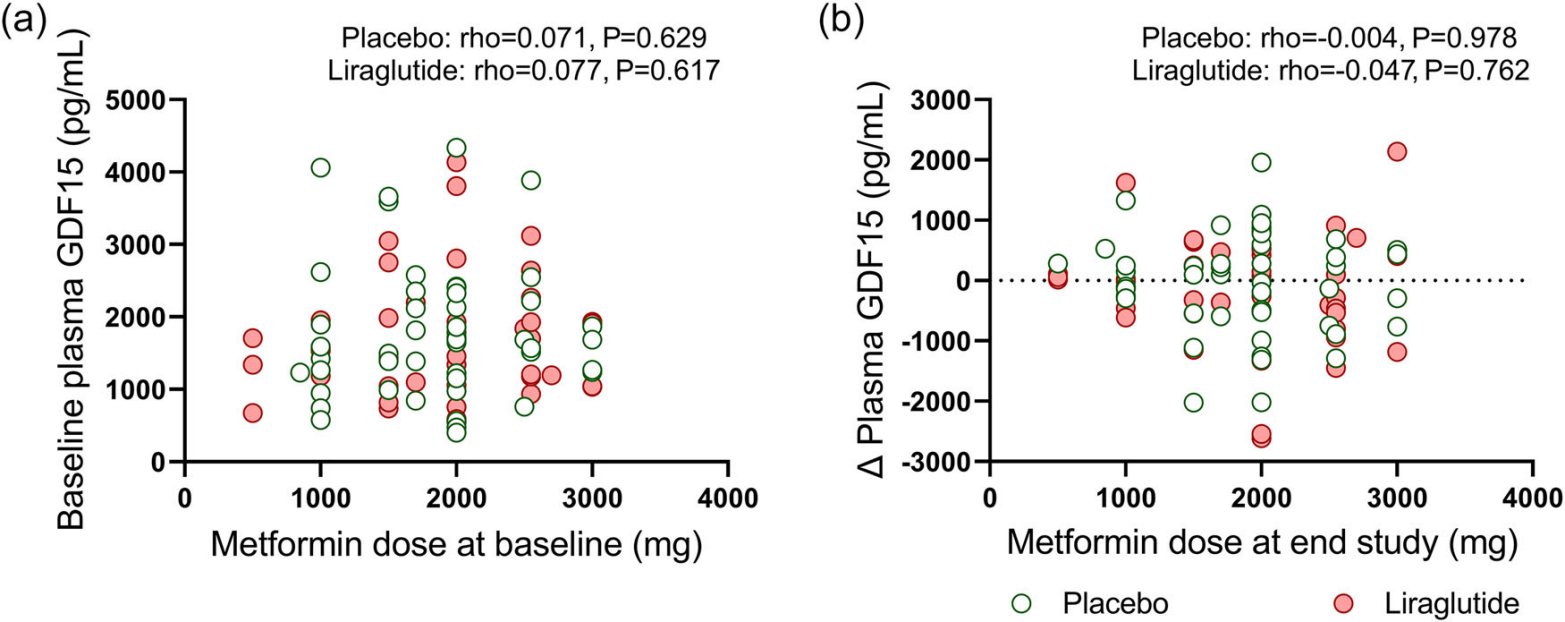
Correlation between metformin dose and change of plasma GDF15. Spearman correlations between the metformin dose at baseline and baseline GDF15 levels (a) and between metformin dose at the end of the study and the change in plasma GDF15 levels (b) in both ethnicities combined after placebo (*n* = 48; green circles) or liraglutide (*n* = 44; red circles). Two participants in the placebo intervention arm did not use metformin at baseline (placebo; *n* = 48), but one participant started metformin during the trial (placebo; *n* = 49). Dots represent individual values.

As previously published, metformin increases plasma GDF15 levels to suppress appetite, thereby inducing weight reduction (Coll et al., 2020; Day et al., 2019). Here we found that the metformin dose was not related to plasma GDF15 levels, either at baseline (liraglutide: rho = 0.077; *P* = 0.617; placebo: rho = 0.071; *P* = 0.629, Figure 8a) or after 26 weeks of treatment (liraglutide: rho = −0.047; *P* = 0.762; placebo: rho = −0.004; *P* = 0.978, Figure 8b).

## 4 DISCUSSION

The current study showed that plasma GDF15 levels were similar in South Asians and Europids with T2DM. Furthermore, 26 weeks of daily treatment with the GLP‐1R agonist liraglutide, compared to placebo, did not modify plasma GDF15 levels in either ethnicity. Additionally, we showed that the change in GDF15 levels did not correlate with the decreases in body weight and changes in HbA_1c_ as induced by liraglutide nor with the use of metformin.

In our study, we observed that plasma GDF15 levels at baseline were similar between South Asians and Europids. While GDF15 levels have not been previously compared between South Asians and Europids, some studies have investigated GDF15 gene expression and circulating levels between different ethnic groups. For example, one study (Rybicki et al., 2021) found that mRNA expression of GDF15 in prostate tissue was lower in African American males compared to American Caucasians who underwent a prostatic biopsy. Another study (Nalado et al., 2020) found that serum GDF15 levels were higher in Black people with chronic kidney disease in South Africa compared to the White racial group with chronic kidney disease. Our study specifically focused on differences in plasma GDF15 levels between Europids and South Asians, a population known for their unfavourable metabolic phenotype and high risk of developing cardiometabolic complications (Anjana et al., 2011; Bakker et al., 2013; Eastwood et al., 2015; McKeigue et al., 1991). GDF15 is a well‐known biomarker for metabolic diseases, with higher GDF15 levels corresponding to metabolic diseases such as atherosclerosis, cardiomyopathies, obesity, insulin resistance, and chronic kidney diseases (Adela & Banerjee, 2015; Berezin, 2016; Hong et al., 2014). Considering the disadvantageous metabolic phenotype of the South Asian population in addition to the previously found differences in the regulation of appetite‐regulating hormones, we hypothesized that GDF15 levels would be different in the South Asian population compared to the Europids. However, we did not find a significant difference in plasma GDF15 levels between South Asians and Europids. A possible explanation for this could be the longer duration of diabetes in South Asians. Metformin is the first‐line pharmacological intervention for patients with T2DM (American Diabetes Association Professional Practice Committee, 2021). Since the South Asian participants in this study had longer T2DM duration than the Europid participants, it is possible that they have used metformin for a longer time than Europids, influencing GDF15 levels in this study. Additionally, GDF15 could already have reached a plateau and did not further increase after liraglutide treatment.

Our finding that 26 weeks of liraglutide treatment did not affect plasma GDF15 levels in either ethnicity suggests that the liraglutide‐induced weight reduction is independent of the GDF15/GFRAL system. This vision is in line with a recent preclinical study showing that the absence of GDF15 or GFRAL signalling did not affect the ability of liraglutide to reduce food intake in mice (Frikke‐Schmidt et al., 2019). There, it was found that while many GFRAL neurons in the area postrema of the mouse and human hindbrain also contained various amounts of GLP‐1R mRNA, the majority of the GLP‐1R neurons did not express GFRAL mRNA. When mice were injected with liraglutide subcutaneously, food intake decreased independent of whether the mice lacked GFRAL or GDF15. In addition, when mice that lacked the GLP1‐R on a whole‐body level were injected subcutaneously with GDF15, their food intake was reduced to the same extent as in mice that did not lack the GLP1R. Of note, when mice were injected with the combination of GDF15 and liraglutide, they had a higher reduction of food intake and weight loss than with injection of either treatment alone, indicating an independent effect of liraglutide on the GDF15/GFRAL system with possible synergetic potential. Furthermore, our data align with a recent human study that appeared during the preparation of our manuscript (Valenzuela‐Vallejo et al., 2022). In that study, GDF15 levels in male and female patients with obesity (*n* = 20) did not change upon treatment with liraglutide for 5 weeks. Since a previous study involving metformin (Coll et al., 2020) showed that plasma GDF15 levels continued to rise after a more extended period of metformin treatment (26, 52 and 78 weeks), 5 weeks of treatment with liraglutide may have been too short to modify plasma GDF15 levels. However, in the current study, in which participants were treated for 26 weeks, plasma GDF15 levels also remained unaffected, demonstrating that an extended treatment period of liraglutide treatment is unable to modify plasma GDF15 levels as well. In addition, we did not find a correlation between the change in GDF15 levels upon liraglutide treatment and the change in weight loss. Altogether, our study supports that the beneficial effects induced by the GLP‐1R agonist liraglutide do not involve the GDF15/GFRAL system.

Taken together, we postulate that the weight‐reducing effect of liraglutide is likely mediated by other systems. GLP‐1 interacts with vagal afferent neurons to transmit gut signals to the hindbrain where it induces satiety, possibly serving as the primary mechanism for satiety induction by GLP‐1 receptor agonism (Owyang & Heldsinger, 2011). This is supported by the attenuated anorexic effect of GLP‐1 agonism when the vagal afferents are denervated (Iwasaki et al., 2018). In addition, other factors such as delayed gastric emptying, influencing fat distribution, and the possible involvement of brown adipose tissue, resulting in enhanced thermogenesis, may contribute to liraglutide‐induced weight loss as well (Duan et al., 2022; Janssen et al., 2020; Marathe et al., 2011). We previously found that treating healthy men with the GLP‐1R agonist exenatide for 12 weeks enhanced glucose uptake by brown adipose tissue, pointing to enhanced brown adipose tissue volume (Janssen et al., 2020). Although our current study points toward an effect of liraglutide on weight loss independent of GDF15, the concept of GDF15 as a potential treatment strategy for people living with obesity remains intriguing. A recent study showed that a long‐acting GDF15 analogue was successful in reducing food intake in rodents and in humans living with overweight and obesity (Benichou et al., 2023).

Although metformin has previously been shown to increase plasma GDF15 levels, in the current study we did not find a correlation between the dose of metformin and plasma GDF15 levels, neither at baseline nor at the end of the intervention period. Although multiple research groups previously described that metformin decreases food intake via increasing plasma GDF15 levels, GDF15 levels were measured up to only 18 months of treatment (Coll et al., 2020; Day et al., 2019). The participants in our study had been living with diabetes ranging from 11 to 19 years. To our knowledge, no long‐term studies have looked at GDF15 levels during prolonged metformin use. Therefore, the GDF15 levels may have reached a plateau or even decreased after long‐term treatment, which could explain why we did not find a correlation between metformin dose and plasma GDF15 levels. In addition, the majority of patients included in the study had T2DM for over 10 years and used other pharmacological therapies, such as insulin and/or sulfonylurea derivates, in addition to metformin. Thus, the presence of polypharmacy resulting from prolonged diabetes duration may also have influenced plasma GDF15 levels in our study. In addition, diabetes‐related complications, such as atherosclerosis development, as well as the presence of obesity itself may have influenced GDF15 levels.

The strengths of our study are the large sample size of 94 participants, with about 50% females. Secondly, we also included patients of South Asian descent, an ethnic population with a high cardiometabolic disease risk, to assess whether ethnic differences exist in plasma GDF15 levels. Moreover, this would give us the opportunity to assess whether liraglutide‐induced effects on GDF15 levels differed between ethnicities. However, this study is not without limitations. We only measured plasma GDF15 levels at two time points: baseline and after 26 weeks of intervention. Therefore, any changes in plasma GDF15 levels within that period could have been missed. Plasma GDF15 is known to be influenced by many factors, such as pharmacological agents (Baek et al., 2001; Sarkar et al., 2022). Almost half of our population received multiple pharmaceutical agents during the intervention period, which could have influenced plasma GDF15 values as well.

In conclusion, this study showed that plasma GDF15 levels were similar in Dutch South Asians and Dutch Europids. Additionally, we observed that 26 weeks of liraglutide treatment does not modify plasma GDF15 levels in either ethnicity. Therefore, we conclude that the GDF15/GFRAL system likely does not play a role in the weight loss induced by liraglutide.

## AUTHOR CONTRIBUTIONS

Conception or design of the work: Carlijn A. Hoekx, Maaike E. Straat, Mariëtte R. Boon, and Borja Martinez‐Tellez. Conception or design and acquisition of data of the original investigation: Maurice B. Bizino, Huub J. van Eyk, Hildebrandus J. Lamb, Johannes W.A. Smit, and Ingrid M. Jazet. Analysis of the data: Carlijn A. Hoekx, Saskia C.A. de Jager, and Borja Martinez‐Tellez. Drafting of the work: Carlijn A. Hoekx. Revising it critically for important intellectual content: Maaike E. Straat, Maurice B. Bizino, Huub J. van Eyk, Hildebrandus J. Lamb, Johannes W.A. Smit, Ingrid M. Jazet, Saskia C.A. de Jager, Mariëtte R. Boon, and Borja Martinez‐Tellez. All authors approved the final version of the manuscript and agree to be accountable for all aspects of the work ensuring that questions related to the part of the work are appropriately investigated and resolved. All persons designated as authors qualify for authorship, and all those who qualify for authorship are listed.

## CONFLICT OF INTEREST

The authors declared no conflict of interest.

## Supporting information

Supplemental Table 1. Baseline characteristics.

## Data Availability

All data reported in this paper will be shared by the lead contact upon reasonable request.
