# Supplementary material for: Growth differentiation factor 15 is not modified after weight loss induced by liraglutide in South Asians and Europids with type 2 diabetes mellitus
Source: Exp Physiol. 2024 Jul 4;109(8):1292–304. doi: 10.1113/EP091815 (PMC11291866; doi:10.1113/EP091815)
Supplement: Supplementary file 1 — Supplemental Table 1. Baseline characteristics. [file EPH-109-1292-s001.docx]

**Supplemental Table 1. Baseline characteristics**

|  | **Europids** | | **South Asians** | | **Combined** | |
| --- | --- | --- | --- | --- | --- | --- |
|  | **Placebo  (*n*=25)** | **Liraglutide  (*n*=22)** | **Placebo  (*n*=25)** | **Liraglutide  (*n*=22)** | **Placebo  (*n*=50)** | **Liraglutide**  **(*n*=44)** |
| **Demographics** | | | | | | |
| **Females, n, %** | *n*=11, 44% | *n*=9, 41% | *n*=14*,* 56% | *n*=14, 64% | *n*=25, 50% | *n*=23, 52% |
| **Age, years** | 58.9 ± 6.7 | 59.9 ± 6.4 | 54.6 ± 9.4 | 55.2 ± 11.1 | 56.7 ± 8.4 | 57.5 ± 9.2 |
| **Diabetes duration, years** | 10.5 ± 6.9 | 10.4 ± 5.0 | 17.0 ± 9.8^#^ | 18.8 ± 10.3^##^ | 13.8 ± 9.0 | 14.6 ± 9.0 |
| **Clinical parameters** | | | | | | |
| **Body weight, kg** | 93.8 ± 12.9 | 98.6 ± 14.1 | 77.8 ± 12.4^###^ | 81.9 ± 11.0^###^ | 85.8 ± 14.9 | 90.3 ± 15.1 |
| **Body length, cm** | 172.4 ± 9.7 | 173.4 ± 7.8 | 164.8 ± 9.4^##^ | 164.3 ± 8.1^###^ | 168.6 ± 10.2 | 168.9 ± 9.1 |
| **BMI, kg/m^2^** | 31.5 ± 3.5 | 32.8 ± 4.3 | 28.6 ± 4.0^##^ | 30.4 ± 3.8 | 30.1 ± 4.0 | 31.6 ± 4.2 |
| **Waist circumference, cm** | 108.4 ± 8.1 | 111.9 ± 9.6 | 98.4 ± 10.1^###^ | 104.1 ± 7.8* ^##^ | 103.4 ± 10.4 | 108.0 ± 9.5* |
| **Hip circumference, cm** | 106.2 ± 6.7 | 108.5 ± 8.5 | 104.0 ± 9.0 | 104.3 ± 7.1 | 105.1 ± 7.9 | 106.4 ± 8.0 |
| **Waist to hip ratio** | 1.0 ± 0.1 | 1.0 ± 0.1 | 0.9 ± 0.1^##^ | 1.0 ± 0.1* | 1.0 ± 0.1 | 1.0 ± 0.1 |
| **Body fat percentage, %** | 36.7 ± 9.0 | 36.7 ± 10.1 | 36.9 ± 9.8 | 37.2 ± 8.4 | 36.8 ± 9.3 | 37.0 ± 9.2 |
| **Subcutaneous adipose tissue, cm^2^** | 330 ± 109 | 367 ± 143 | 326 ± 141 | 316 ± 97 | 328 ± 125 | 341 ± 123 |
| **Visceral adipose tissue, cm^2^** | 200 ± 62 | 211 ± 88 | 149 ± 49^##^ | 187 ± 57* | 174 ± 61 | 199 ± 74 |
| **Visceral/subcutaneous adipose tissue ratio** | 0.7 ± 0.3 | 0.7 ± 0.4 | 0.5 ± 0.3 | 0.7 ± 0.3 | 0.6 ± 0.3 | 0.7 ± 0.3 |
| **Epicardial adipose tissue, cm^2^** | 9.6 ± 4.1 | 8.9 ± 4.4 | 9.1 ± 2.7 | 10.4 ± 3.2 | 9.3 ± 3.4 | 9.6 ± 3.9 |
| **Paracardial adipose tissue, cm^2^** | 20.6 ± 10.0 | 25.8 ± 11.2 | 9.0 ± 4.5^###^ | 12.3 ± 4.4* ^###^ | 14.4 ± 9.5 | 19.1 ± 10.8* |
| **Pericardial adipose tissue, cm^2^** | 30.2 ± 12.3 | 34.7 ± 13.7 | 18.2 ± 5.6^###^ | 22.7 ± 6.5* ^##^ | 23.9 ± 11.1 | 28.7 ± 12.2* |
| **HbA_1c_, mmol/mol** | 64.6 ± 10.3 | 66.5 ± 11.7 | 70.5 ± 12.1 | 64.8 ± 9.7 | 67.5 ± 11.5 | 65.7 ± 10.7 |
| **Total cholesterol, mmol/L** | 4.8 ± 1.0 | 4.9 ± 1.0 | 4.5 ± 1.1 | 4.0 ± 0.7^##^ | 4.6 ± 1.1 | 4.4 ± 1.0 |
| **HDL-C, mmol/L** | 1.3 ± 0.4 | 1.2 ± 0.3 | 1.2 ± 0.3 | 1.2 ± 0.3 | 1.3 ± 0.3 | 1.2 ± 0.3 |
| **LDL-C, mmol/L** | 2.5 ± 0.9 | 2.6 ± 0.9 | 2.2 ± 1.0 | 2.0 ± 0.7^##^ | 2.4 ± 1.0 | 2.4 ± 0.9 |
| **Diabetes medication** | | | | | | |
| **Metformin use, n, %** | *n*=25, 100% | *n*=22, 100% | *n*=23, 92% | *n*=22, 100% | *n*=48, 96% | *n*=44, 100 % |
| **Metformin, mg/day** | 1982 ± 553 | 2093 ± 700 | 1728 ± 643 | 1750 ± 665 | 1860 ± 605 | 1922 ± 697 |
| **Sulfonylurea, n, %** | *n*=8, 32% | *n*=6, 27% | *n*=5, 20% | *n*=3, 14% | *n*=13, 26% | *n*=9, 21% |
| **Insulin use, n, %** | *n*=16, 64% | *n*=14, 64% | *n=*19, 76% | *n*=17, 77% | *n*=35, 70% | *n*=31, 71% |

Adapted from the original data from treatment in Europids (Bizino et al., 2019). and South Asians (van Eyk et al., 2019). Two Europids were not included in the analyses since they discontinued treatment. BMI, body mass index; HbA_1c_, hemoglobin A1c; HDL-C, high-density lipoprotein-cholesterol; LDL-C, low-density lipoprotein-cholesterol; Asterisk signs (*) indicate significant differences between treatments within a specific ethnicity, and hash signs (^#^) indicate significant differences between ethnicities within a specific treatment group. *P < 0.05, ^#^P < 0.05, ^##^P < 0.01, ^###^P < 0.001. Data are presented as mean ± standard deviation.
